# Stereotactic Ventriculostomy Guide

**DOI:** 10.7759/cureus.21089

**Published:** 2022-01-10

**Authors:** Arun A Patil, Ashis Chand, Deepak K Pandey, Amelia Simmons, Thomas T Nilles-Melchert

**Affiliations:** 1 Surgery, Creighton University School of Medicine, Omaha, USA; 2 Neurosurgery, St John's Medical Academy, Bangalore, IND; 3 Orthopedics - Spine, Less Exposure Surgery (LES) Institute, Malden, USA

**Keywords:** degree of freedom - df, mr-magnetic resonance, ct-computed tomography, cm-centimeter, cerebral ventricle drainage, hydrocephalus, small ventricles, stereotactic, ventriculostomy guide

## Abstract

Introduction

Based on the anatomy of the frontal horn, a stereotactic ventriculostomy guidance system that does not need an elaborate setup and is suitable for ventricles of all sizes was developed. The objective of this paper is to describe this system and present the results of a cadaveric study in which this system was used.

Method

The system has a midline-based plate that is contoured to snugly fit the top of the head. It has two probe holders, one on each side at pre-set angles in coronal and sagittal planes, which enables the probe holders to point at the foramen of Monro. A cadaver study was done on eight donors. First, using the guidance system a 1.4 mm endoscope was inserted into the right frontal horn through a twist drill hole. Next, the scope was inserted into the right frontal horn on the same donors using the freehand method.

Result

With the guide, all eight ventricles were entered into on the first trial, and the foramen of Monro was visible end-on. With freehand technique: six ventricles were entered on the first try; however, the foramen of Monro was visible end-on only in one. In the other two, two to three attempts were needed. The guide facilitated 100% visibility for the end-on visibility of the foramen of Monro upon insertion and the results were statistically significant with t=7, df=7, p-value=0.000106.

Conclusion

This is a simple system, which is easy to use. The cadaveric study showed a high degree of accuracy to access the ventricles. The data shows significant improvement compared to the freehand technique.

## Introduction

The free-hand approach to ventricular catheter placement is a common surgical procedure, which is often performed at the bedside. Though the procedure is straightforward and easy to perform, there is a small but significant percentage of failure rate to enter the intended target on the first attempt. Furthermore, if the ventricles are small the failure rate is higher [1-3]. To solve this problem, surgeons have used optical navigation, electromagnetic navigation, dynamic reference frame, ultrasound imaging, computer assistance system, and smartphone [4-5]. However, these systems either take a longer time to set up or introduce inaccuracy due to varying skull contours. Since the ventricular catheter placement procedures are often done at the bedside under emergency situations and under intense time pressure, a system that is simple and quick to set up is needed.

A tripod-shaped guidance system has been described, which enables intracranial entry of the catheter perpendicular to the skull [6]. However, its accuracy is affected by the contour of the calvarium [7]. Another system described is the Thomale guide [8], a protractor-conductor with a stand, which guides the catheter into the ventricle. The angle and the entry point are determined using prior CT or MRI images and special software. It does need set-up time. In addition, the system sits on the lateral surface of the skull, which can introduce inaccuracy. Furthermore, it has no preset angle for the sagittal plane. The sagittal plane tilt, therefore, is performed using the free-hand method.

The authors, in this paper, describe a new stereotactic guidance system that has preset angles of tilt of the probe holders in the frontal and coronal planes at preset distances. This enables the operator to aim at the foramen of Monro and reach the smallest possible ventricle via frontal approach. These angles are selected based on the evaluation of 15 MRI scans of 15 different subjects (patients) with small and medium-sized ventricles. The base plate of the system is placed in the midline on the top of the head and is contoured, both in the coronal and sagittal planes. This enables the operator to snugly attach the system to the scalp surface.

## Materials and methods

Description of the system

**Figure 1:**
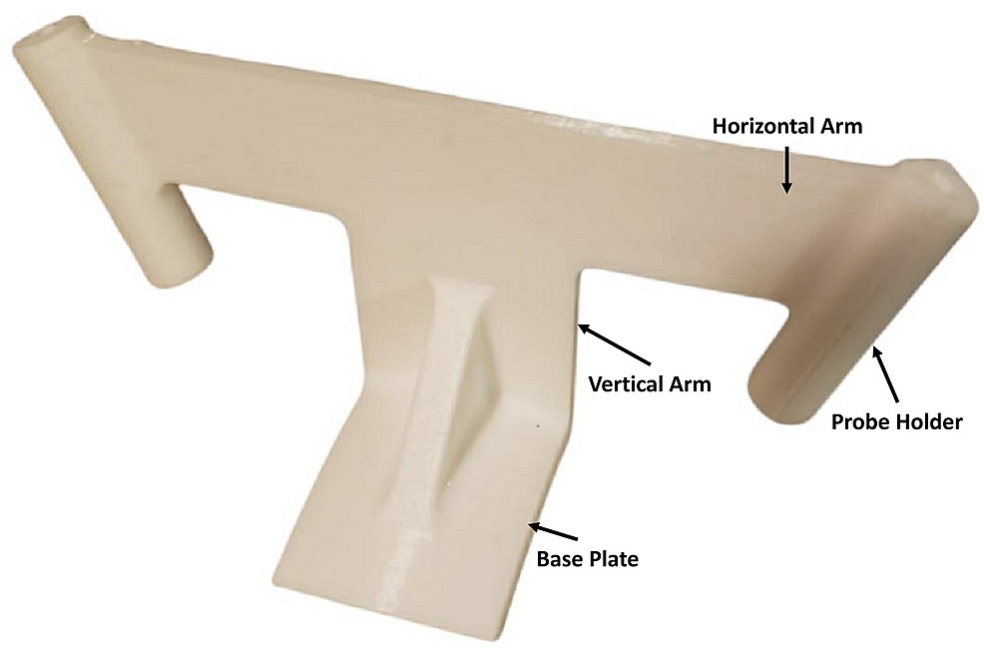
3D printed model of the stereotactic ventriculostomy guide showing key features

The ventriculostomy guide (system) consists of a base plate, a vertical arm, two probe holders, and a horizontal arm (Figure 1).

**Figure 2:**
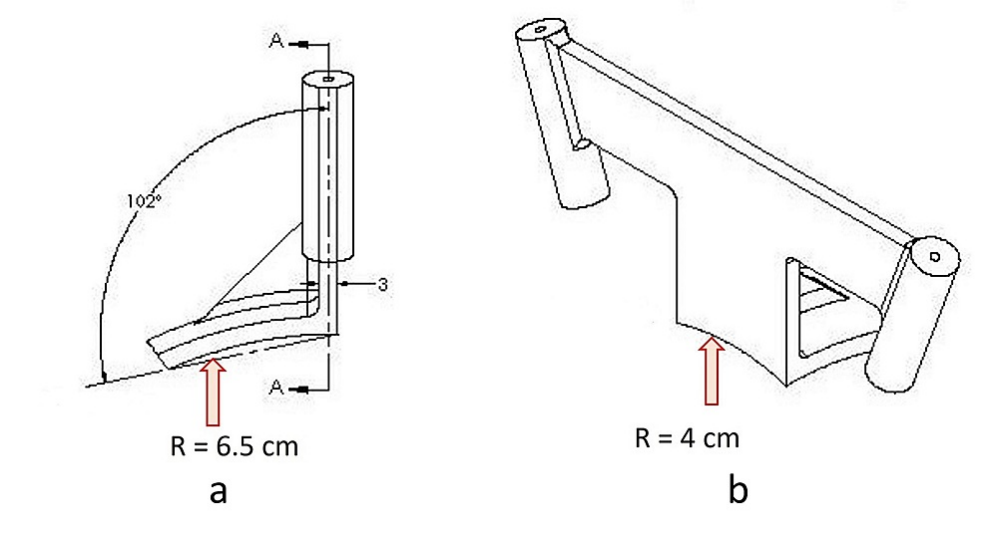
a - Diagram showing curvature in the base plate (arrow) in the sagittal plane; b - Diagram showing curvature in the base plate (arrow) in the coronal plane

The base plate is curved both in the sagittal (Figure 2a) and coronal (Figure 2b) planes, enabling it to contour and snugly fit the top of the head.

**Figure 3:**
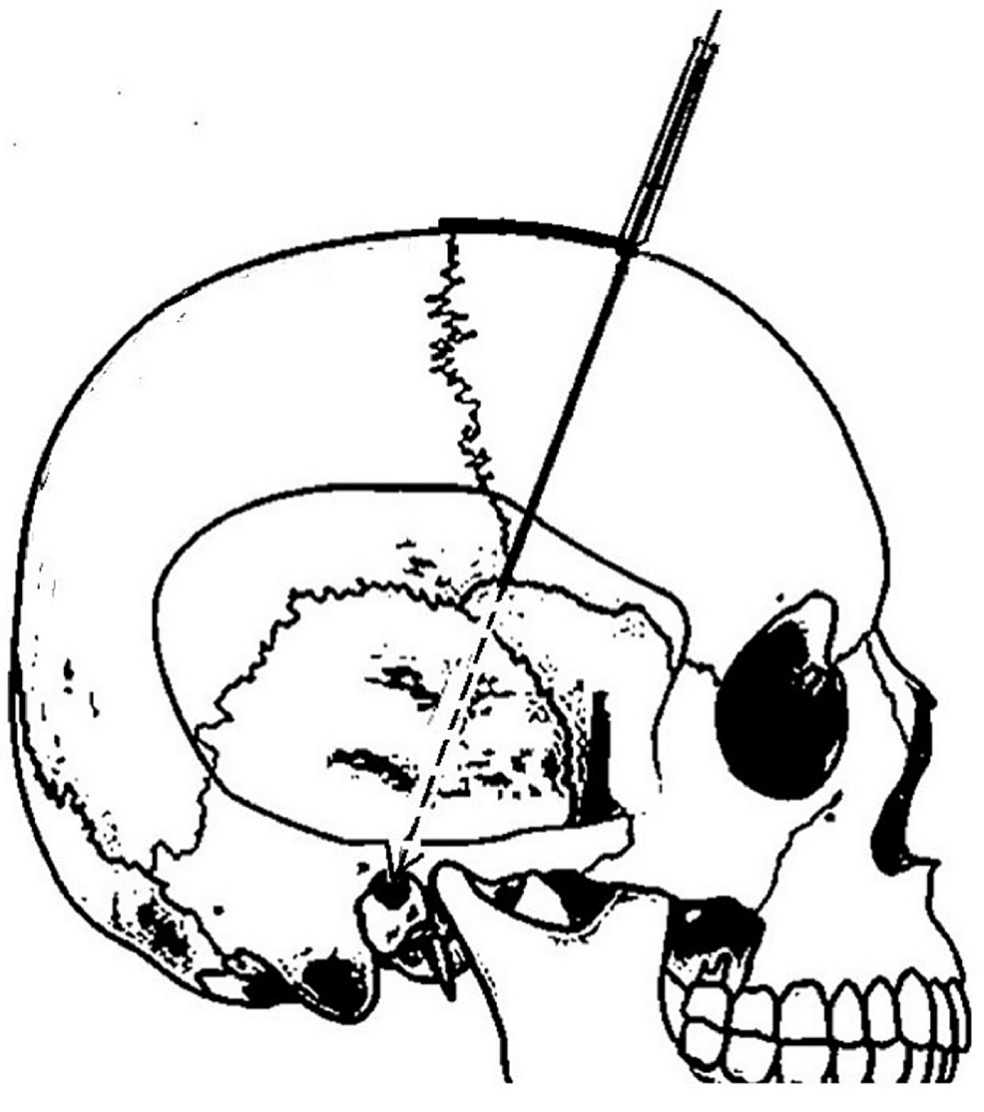
Shows the template of the scope on the top of the head on lateral view of skull image with the probe holder in line with the external auditory meatus

**Figure 4:**
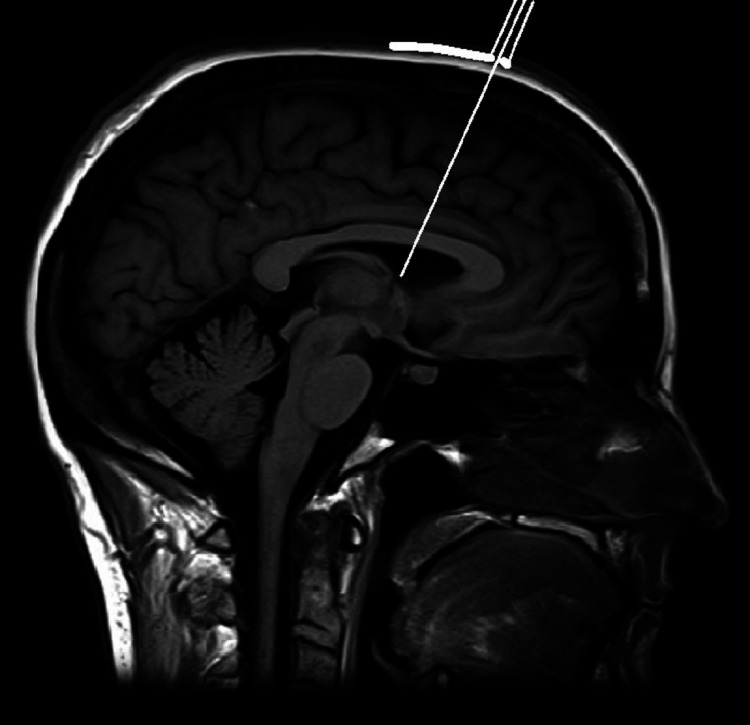
Shows the template of the scope on the top of the head on sagittal MR image with the probe holder aimed at the foramen of Monro

The base plate has a radius of 6.5 cm in the sagittal plane and a radius of 4 cm in the coronal plane. The bottom of the base plate has an adhesive coating to secure the base plate to the scalp. The midline of the anterior end of the baseplate has a vertical arm connecting to a horizontal arm containing two probe holders at its lateral ends (Figure 1). The probe holders have an inner diameter of 3.5 mm. Depending on the outer diameters of the catheter used, tubular inserts with smaller inner diameters are available. The vertical arm in the sagittal plane has an angle of inclination of 102 degrees with a straight line connecting the two ends of the sagittal curvature of the base plate (Figure 2a). This directs the probes holder in the sagittal plane towards the external auditory meatus (Figure 3) and foramen of Monro (Figure 4) when the anterior end of the vertical arm is 13 cm posterior to the nasion.

**Figure 5:**
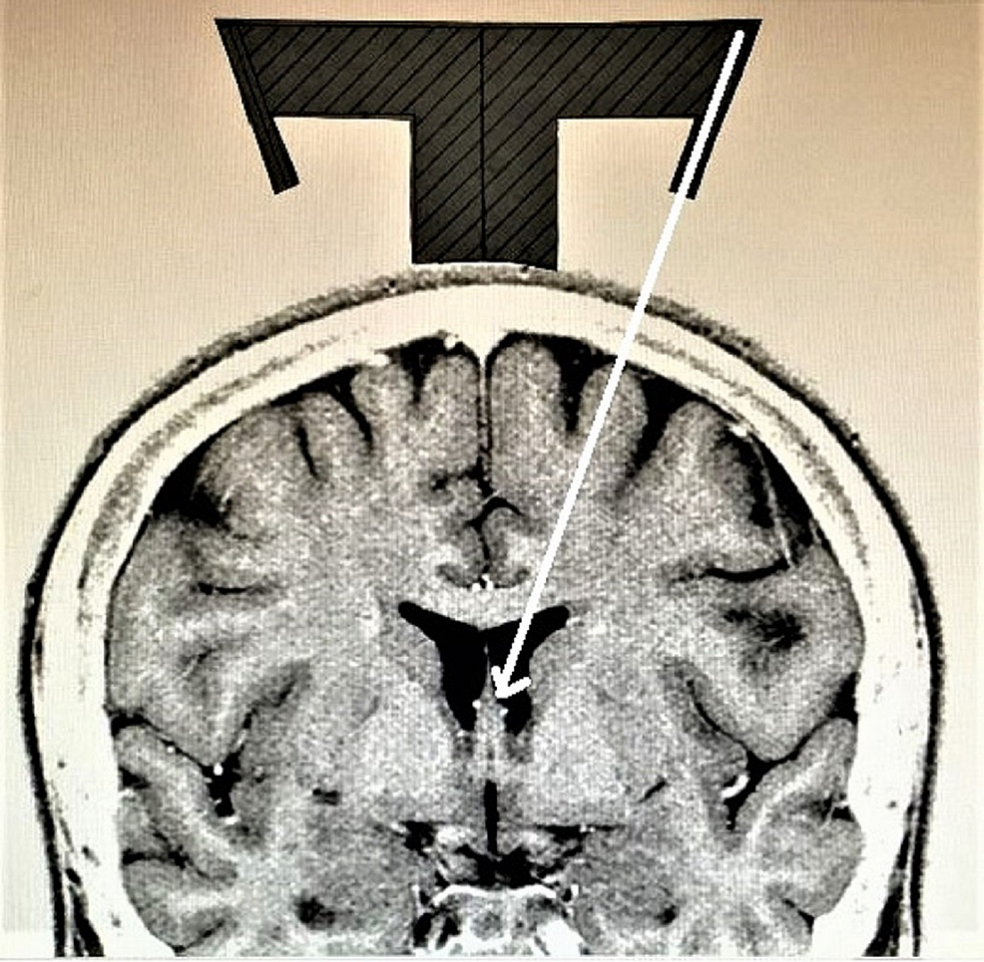
Shows the template of the guide on top of the head on a coronal MR image with the probe holder aimed towards the foramen of Monro

In the coronal plane, the probe holders are inclined medially with an angle of 70 degrees horizontal (Figure 5). The above-mentioned angles of inclinations enable the probe holder to aim medially and down towards the foramen of Monro. The trajectory from the probe holder to the target touches the outer surface of the skull 3 cm from the midline. Therefore, the entry hole in the skull is 3 cm from the midline.

The system has templates for both sagittal (Figure 4) and coronal (Figure 5) planes that can be laid on MR images to confirm accuracy prior to the procedure.

Given below is the method and steps to use the guide for the ventriculostomy:

Step 1

This step is needed only if the ventricles are extremely small, or there is a midline shift, or the head is small or irregular in contour. Review the CT or MR image in the coronal plane showing the frontal horn and third ventricle and magnify it to life-size. To obtain a life-size image: (a) use the measure distance mode to measure the right to left distance on the coronal image at its greatest width; (b) then put a ruler on the line of measurement and change the magnification of the image until the ruler shows the same distance as the measured distance on the computer.

The coronal template is then placed on the top of the head on the coronal image to confirm that the probe holder is aiming into the ventricle towards the foramen of Monro (Figure 5). The same process can be done in the sagittal plane (Figure 4). In cases where there is a midline shift, the template can be moved to the side of the shift until the probe holder aims at the target. The distance moved is noted and used for placement of the base plate on the scalp. 

In pediatric patients, the template is slowly moved to a higher height from the scalp until the probe holder aims at the target. The distance of the lift is noted, and an appropriate thickness lift is attached to the bottom of the base plate. 

Step 2

Midline is marked on the skin from the nasion posteriorly to the top of the head. A point, 13 cm posterior to the nasion, is marked. The ventriculostomy guide is placed on the head in the midline with the anterior end of the base plate at the 13 cm mark. It is stabilized in this position by the adhesive pad and can also be done manually by an assistant. A drill bit is first inserted up to the skin through the probe holder, and that point is marked on the skin. After a local anesthetic is injected, a small incision is made at a point. A small self-retaining retractor is put to expose the skull surface. A hole is drilled through the skull with the drill bit guided through the probe holder. The dura is opened.

Step 3

The distance from the skull surface (at the entry point) to the target in the ventricle is measured on a coronal CT or MR image. This distance is marked on the catheter to indicate the limit of catheter insertion from the skull surface. An insert that would snugly accommodate the ventricular catheter is placed in the probe holder. The catheter is then inserted into the ventricle.

Cadaver study

Cadaver studies were performed on eight formalin-fixed cadavers. Ethics approval and consent to participate were not needed. The families of the donors had consented to use the donors for teaching and research purposes. Consent for publication was not applicable.

**Figure 6:**
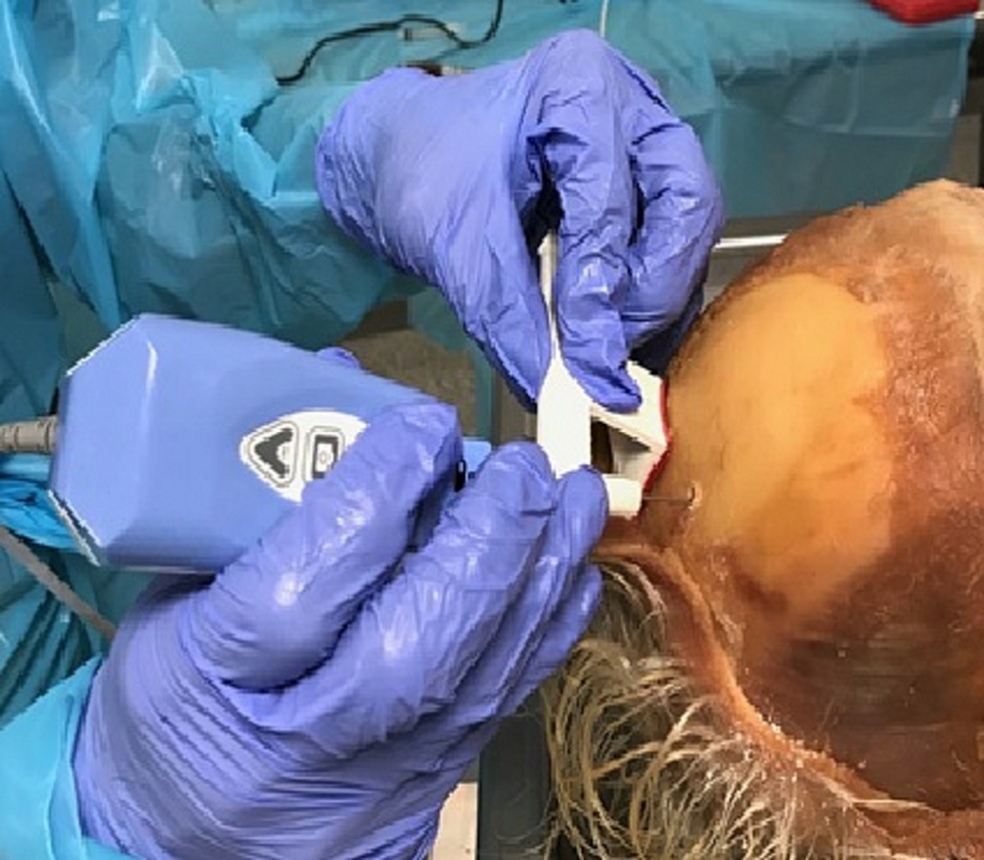
Image shows guide being used to insert the scope into the ventricle.

A 1.4 mm diameter MIDA scope (INTRA VU, Redwood City, CA) was inserted (Figure 6) through the brain into the ventricle in all eight donors using the ventriculostomy guide per the technique described above.

**Figure 7:**
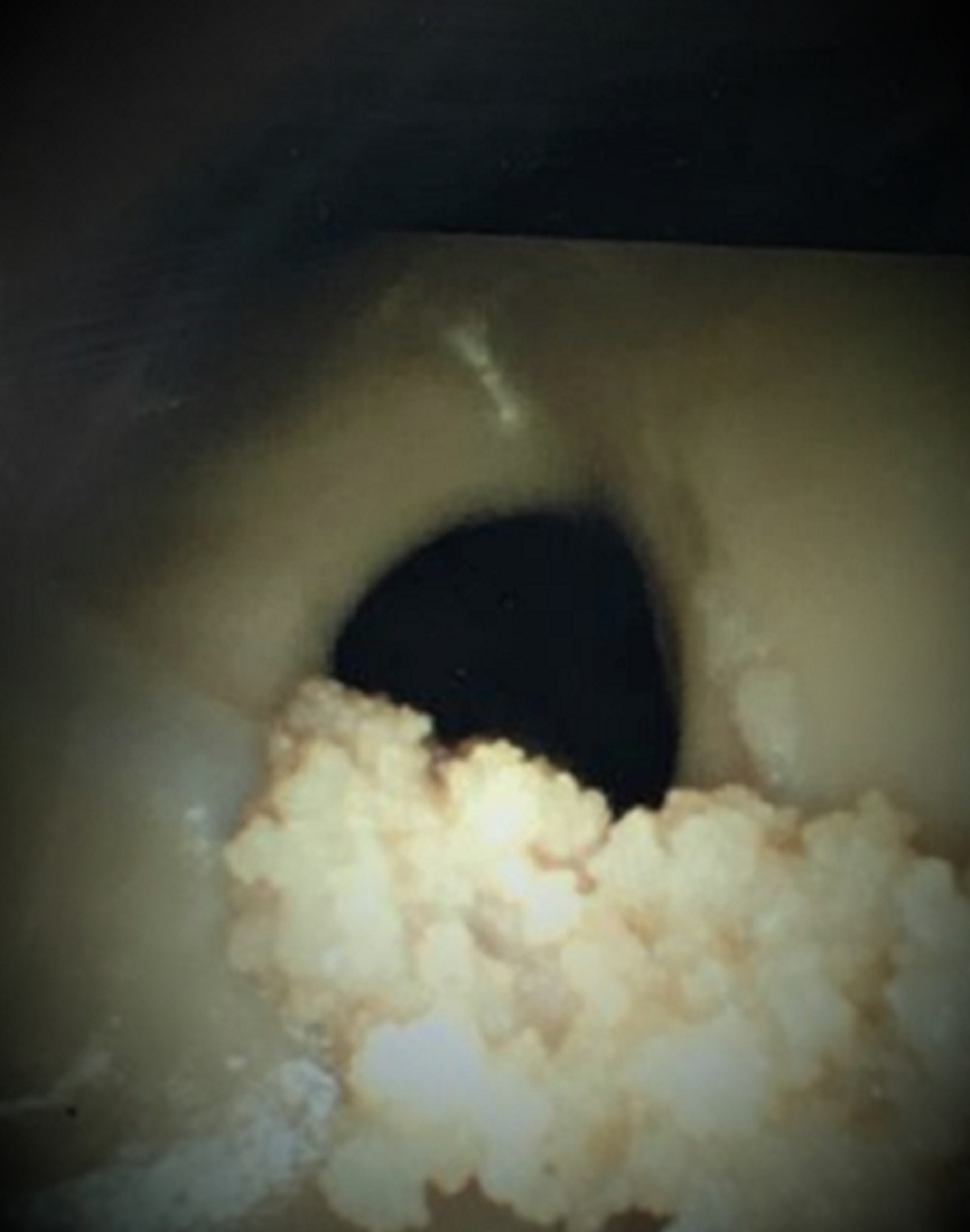
Shows end-on view of the foramen of Monro through the endoscope

The procedure was then repeated on the same eight donors using the freehand method by aiming the catheter to the medial canthus of the ipsilateral eye in the coronal plane and toward the external auditory meatus in the sagittal plane. The procedures were done by a neurosurgeon with 50 years of experience. The number of attempts taken to enter the ventricle was recorded along with straight-ahead visibility of foramen of Monro (Figure 7).

Data and statistical analysis were performed by the Microsoft Excel data analysis tool (Microsoft Corporation, Redmond, WA). A t-test: two-sample assuming unequal variances was conducted to analyze the visibility of the foramen of Monro and the entry of the scope into the ventricle via the freehand and guided approaches.

## Results

Ventriculostomy using the guide in the study

In all eight cases, the ventricles were entered into on the first trial and the foramen of Monro was visible end-on. Furthermore, when the scope was advanced deeper, it entered the foramen of Monro with every attempt.

Ventriculostomy using the freehand technique

In six donors, the ventricle was entered on the first trial; however, the foramen of Monro was visible end-on only in one. In one donor, two tries were required to enter the ventricle and in one donor, three trials were needed to enter the ventricle.

With the stereotactic guide presented in this study, it took only one attempt to enter the ventricle as opposed to an average of 1.38 attempts via freehand. The result for catheter insertion using the guided versus free-hand technique was non-significant with t = -1.42557, df = 7, p-value = 0.0985.

With the stereotactic guide, the visibility of the foramen of Monro end-on was in 100% of the time while inserting the scope into the ventricle as opposed to only 13% of the time with the freehand technique. The result was found to be statistically significant with t = 7, df = 7, and p-value = 0.000106. The results were considered significant for p-value < 0.05 and analysis was performed by the Microsoft Excel data analysis tool.

In summary, the stereotactic guide presented in this study was found to be 38% superior (1 attempt via guide vs. 1.38 attempts via freehand) compared to the freehand technique when entering the ventricles, and 87% superior (100% of the times via guide vs. 13% of the times by freehand) compared to the freehand technique for the end-on visibility of the foramen of Monro.

## Discussion

Though ventriculostomy is a common procedure and easy to perform, it is not unusual to need more than one trial to enter the ventricle [1-3]. Obviously, one can easily use a navigation system in such cases. This, however, becomes difficult in an emergency situation, which is usually done at the bedside. Therefore, a guidance system that is simple and effective is needed. There are two prior ventricular guides described [6-8]; however, they have their shortcomings, which have been mentioned in the background section of this paper.

Lind et al. studied the difficulty of ventriculostomy extensively and concluded that the major difficulty is the coronal plane [9]. They also suggested optimal angles in coronal and sagittal planes to reach the ventricle. Unfortunately, the angles used to approach the ventricle can be inaccurate because there is no solid horizontal plane to measure the angle, and angles determined on surfaces with slopes (skull) can cause inaccuracies.

The area of the head with a fairly common surface contour is in the midline and just anterior to the coronal suture. In the presented guide, therefore, the base plate is placed in the midline around the coronal suture. Furthermore, the base plate has curvatures, in coronal and sagittal planes to make it sit snugly on the top of the head. In addition, the probe holder has preset angles in coronal and sagittal planes. The radii of the curves on the base plate and the preset angles of the probe holders are based on studies of 15 MR images.

The current system is not currently labeled and approved for use. It is designed with preset distances, curves, and angles to make it suitable for all sizes of ventricles. It has two probe holders, one for each side, and they have pre-set angles both in the coronal and sagittal planes. Although the margin of error to tap the frontal horn is greater in the sagittal and axial planes because the height and length of septum pellucidum are 12-15 mm and 50-52 mm respectively, it is small in the coronal plane because the size in this plane can be as small as only a slit. Furthermore, the height of the septum pellucidum is greatest at the level of the foramen of Monro. Therefore, the angle setting for the coronal plane was chosen to aim towards the midline at the foramen Monro. Furthermore, though the foramen of Monro can increase in size due to hydrocephalus its location is fairly constant.

Conventionally, the entry point on the skull for ventriculostomy is 3 cm from the midline. Therefore, the height of the horizontal arm was so chosen that the trajectory will touch the head 3 cm from the midline (Figure 6). Prior to manufacturing the device, thick paper templates were made for the coronal and sagittal planes and tested on 15 MR images with small and mid-size ventricles. In each one of these studies, the foramen of Monro was reached.

The system also has templates for its application, both in the coronal and sagittal planes. The operator can use the template to confirm the accuracy of the trajectory. Furthermore, if there is a midline shift, the base plate can be moved to the side of the shift. The operator can determine the correction needed using the template. Similarly, in pediatric patients, correction can be made for the size of the head by putting an attachment under the base plate to raise the height. The template can be used to determine the thickness of the attachment.

Errors can occur with this system if the base plate is not exactly in the midline, or there is a movement of the base plate during the procedure. Therefore, although the pad under the base plate has adhesive to stabilize it on the scalp, it is important also to manually stabilize it.

In the cadaver study, the size of the ventricles was unknown. However, the study still shows that the guide is accurate for small targets because the foramen of Monro (which is a small target) was reached during each attempt. The reach of the foramen of Monro with the guide was 100% for the first try while with the freehand method, it was 13%. The results were statistically significant.

## Conclusions

In conclusion, we have described a new ventriculostomy guide. The system has pre-set angles for the probe holder, both in the coronal and sagittal planes. The base plate also has curvature in the sagittal and coronal planes to contour the guide and make it snugly fit the surface of the calvarium. The guide is easy to use and effective as demonstrated by the study. This study showed excellent accuracy for a small medially located target within the ventricle using the guide.
